# 759. Gaps on Lyme Disease Knowledge Among Healthcare Providers in an Endemic Area: a Call for Educational Programs

**DOI:** 10.1093/ofid/ofad500.820

**Published:** 2023-11-27

**Authors:** E V A N GARY, Pooja Lamba, Andrew S Handel, Miguel A Saldivar, Luis A Marcos

**Affiliations:** STONY BROOK UNIVERSITY, Syosset, New York; Stony Brook University, Stony Brook, New York; Stony Brook Children's Hospital, Stony Brook, New York; SUNY Stony Brook University Hospital, Stony Brook, NY; Stony Brook University, Stony Brook, New York

## Abstract

**Background:**

Lyme disease (LD) is hyperendemic in Long Island, NY. Identifying the practices of healthcare providers regarding LD epidemiology, clinical features, diagnosis, and treatment is crucial to continue improving patient care. Our study aims to assess the general understanding of LD among front line healthcare providers in order to identify knowledge gaps and produce targeted educational materials.

**Methods:**

A 20-question IRB-approved online survey on LD prevention, diagnosis, and treatment practices based on IDSA national multidisciplinary LD guidelines was developed. From January to April 2023, this survey was sent to front line providers including physicians, residents, nurse practitioners, and physician assistants at Stony Brook Medicine. A p-value < 0.05 was considered significant using SPSS software.

IRB-approved survey for Lyme disease.

**Figure:**
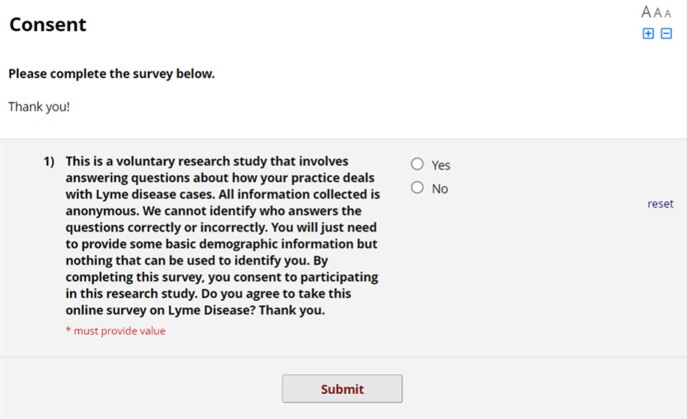

**Results:**

Out of 140 respondents, 93 (66%) fully completed the survey (72% female). Regarding years in training, 53 (57%) reported being in practice for >5 years post-training, 15 (16%) were 1-5 years post-training, and 25 (27%) were still in training. Participants practiced the following specialties: Emergency Medicine 25% (n=23), Internal Medicine (IM) 15% (n=14), Pediatrics (Ped) 54% (n=50), and combined IM/Ped 6 % (n=6). Regarding knowledge of LD: 69% (n=64) of participants correctly identified the tick associated with LD, 48% (n=45) knew the correct description of erythema migrans, 75% (n=70) correctly identified the guideline recommendation for single dose doxycycline prophylaxis after a high-risk tick bite, 68% (n=63) offer single dose doxycycline prophylaxis after any tick bite, 35% (n=33) answered that a 7-10 day course of doxycycline for prophylaxis is indicated after a high-risk bite and only 3% (n=3) correctly identified the CDC-recommended testing strategies for the diagnosis of LD. In regression models, age, gender, specialty, or years in practice did not improve knowledge in these variables.

Demographics table.

**Figure:**
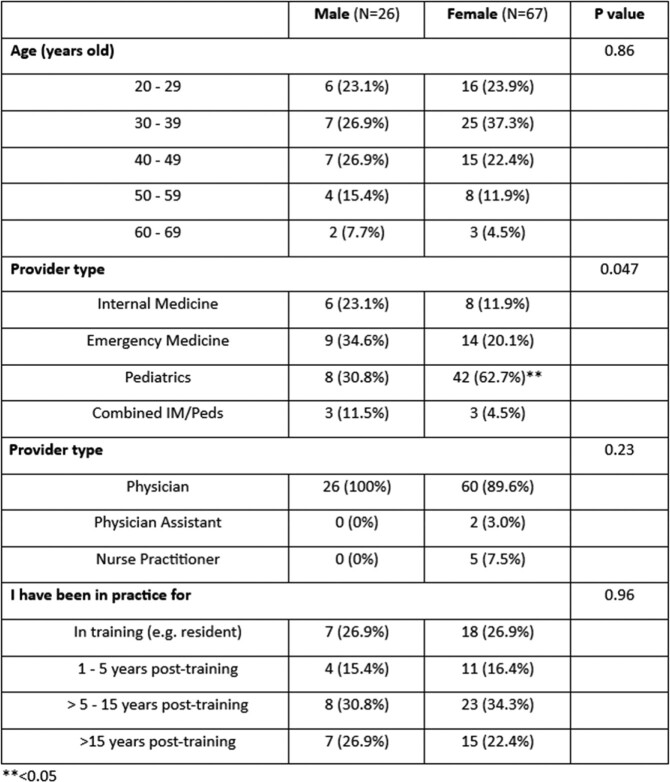

**Conclusion:**

Our pilot study found numerous important knowledge gaps among the surveyed providers regarding the current recommendations for prevention, diagnosis and treatment of LD. Based on these findings, we plan to create targeted educational efforts in order to improve mainly two areas on LD: diagnosis and management.

**Disclosures:**

**All Authors**: No reported disclosures

